# Understanding User Behavior Through the Use of Unsupervised Anomaly Detection: Proof of Concept Using Internet of Things Smart Home Thermostat Data for Improving Public Health Surveillance

**DOI:** 10.2196/21209

**Published:** 2020-11-13

**Authors:** Niloofar Jalali, Kirti Sundar Sahu, Arlene Oetomo, Plinio Pelegrini Morita

**Affiliations:** 1 School of Public Health and Health Systems Faculty of Applied Health Sciences University of Waterloo Waterloo, ON Canada; 2 Institute of Health Policy, Management, and Evaluation University of Toronto Toronto, ON Canada; 3 Department of Systems Design Engineering University of Waterloo Waterloo, ON Canada; 4 eHealth Innovation Techna Institute University Health Network Toronto, ON Canada

**Keywords:** public health, IoT, anomaly detection, behavioral monitoring, deep learning, variational autoencoder, LSTM

## Abstract

**Background:**

One of the main concerns of public health surveillance is to preserve the physical and mental health of older adults while supporting their independence and privacy. On the other hand, to better assist those individuals with essential health care services in the event of an emergency, their regular activities should be monitored. Internet of Things (IoT) sensors may be employed to track the sequence of activities of individuals via ambient sensors, providing real-time insights on daily activity patterns and easy access to the data through the connected ecosystem. Previous surveys to identify the regular activity patterns of older adults were deficient in the limited number of participants, short period of activity tracking, and high reliance on predefined normal activity.

**Objective:**

The objective of this study was to overcome the aforementioned challenges by performing a pilot study to evaluate the utilization of large-scale data from smart home thermostats that collect the motion status of individuals for every 5-minute interval over a long period of time.

**Methods:**

From a large-scale dataset, we selected a group of 30 households who met the inclusion criteria (having at least 8 sensors, being connected to the system for at least 355 days in 2018, and having up to 4 occupants). The indoor activity patterns were captured through motion sensors. We used the unsupervised, time-based, deep neural-network architecture long short-term memory-variational autoencoder to identify the regular activity pattern for each household on 2 time scales: annual and weekday. The results were validated using 2019 records. The area under the curve as well as loss in 2018 were compatible with the 2019 schedule. Daily abnormal behaviors were identified based on deviation from the regular activity model.

**Results:**

The utilization of this approach not only enabled us to identify the regular activity pattern for each household but also provided other insights by assessing sleep behavior using the sleep time and wake-up time. We could also compare the average time individuals spent at home for the different days of the week. From our study sample, there was a significant difference in the time individuals spent indoors during the weekend versus on weekdays.

**Conclusions:**

This approach could enhance individual health monitoring as well as public health surveillance. It provides a potentially nonobtrusive tool to assist public health officials and governments in policy development and emergency personnel in the event of an emergency by measuring indoor behavior while preserving privacy and using existing commercially available thermostat equipment.

## Introduction

The Internet of Things (IoT) is a network of sensors that is integrated with physical devices and other elements to allow objects to become intelligent and interact with humans [1]. The utilization of IoT in health care is growing dramatically, especially in the areas of behavioral monitoring, welfare interventions, and incident notifications [2]. The IoT involves different types of information such as action, movements, and location, as well as physiological monitoring such as gait, heart rate, blood pressure, and stress [3]. The idea of implementing sensors to monitor one’s health status and recognize activity patterns of individuals was initiated by mounting a variety of sensors on the human body [4] as well as smartphones [5] to capture different movements and other health information. In addition to wearable sensors, ambient sensors have also been utilized to build smart homes. The core feature of a smart home is activity recognition (such as watching TV, cooking meals, and sitting on the sofa) that classifies the collected data into well-defined movements [6,7]. This can be a good indicator for predicting normal and abnormal behaviors [6], as well as recognizing diseases and injuries [8,9]. As elderly people are likely to face difficulties with chronic diseases and other issues that accompany aging, a smart home could provide support and enable elderly individuals to live independently, as well as to provide immediate health care services in the event of an injury and other physical or mental health complications [2,6,10-12]. Using smart home technology would also diminish the significant burden and cost of providing long-term care services for supporting older adults that often falls on our health care systems [13-18].

In the context of smart homes, diverse approaches have been implemented to collect data from sensors and recognize different types of activities. In some studies, the activity patterns of individuals are identified by integrating wearable and ambient sensors [19,20]. In others, the interaction of humans and objects is detected through sensory-based devices [10,21,22], and the activity pattern is recognized through the sequence of those interactions [10,23].

Different supervised machine learning and deep learning models have been utilized to detect anomalies. In some approaches, participants self-labeled their activities, and normal and abnormal behaviors were identified by a supervised classification model [6]. In other studies, regular activity patterns are identified through the sequence of time-stamped events. Deviation from those patterns identified abnormal activities [22,24-27], using methods such as clustering of time-stamped events with the deep belief network feature-extraction method [21], Hidden Markov Models [22], and graph-based networks [26].

Although previous approaches towards behavioral monitoring using smart home technology were focused on collecting detailed information about health status and specific activity patterns of individuals, they ultimately proved to be obstructive, as they were limited to a small number of devices, dependent on participants to report the predefined tasks, time-consuming, and not generalizable for other smart homes [25].

Therefore, to tackle these challenges, we implemented a novel, unobstructive strategy to identify abnormal activity patterns of individuals using an ecobee smart-home thermostat. We hypothesized that, through the use of ecobee’s remote sensor data, it would be possible to create models that represent typical user behaviors (eg, sleep time, wake-up time, and average time spent at home), providing the public health community with a novel tool that could identify when abnormal patterns are identified in daily user behavior. The key advantages of this approach are that we can leverage population-level data over a long period while preserving the privacy of individuals. We also implemented an unsupervised neural network for detecting anomalous activity.

## Methods

### Data

In this study, we used Donate Your Data datasets from ecobee, a smart-home thermostat manufacturer from Canada. The data are donated by households that consented to share their anonymized data to conduct research while their privacy is preserved [28]. Around 98% of the users are located in North America [29]. We selected a subset of 30 households that had at least 8 passive infrared embedded sensors and had been online for at least 355 days of 2018. The data collected by the sensors are transmitted to the thermostat base. The thermostat base can support up to 32 sensors. The data are reported in 5-minute intervals. To reduce the noise in the signal, the time window was extended from 5-minute to 30-minute intervals. A similar approach has been utilized by Huchuk et al [29] and Kleiminger et al [30]. For every 30-minute interval, motion status was determined on the basis of the sum of activation from all the sensors (if motion was captured, the sensor value changed from 0 to 1). We identified a positive motion state if the activation of at least 1 sensor lasted for 20 minutes or a minimum of 4 sensors captured a movement for a 5-minute interval each (the sum of activations during a 30-minute interval was ≥4). Therefore, each day is represented by a binary time series that identifies the motion state for the 48 time intervals during a 24-hour period.

### Model

**Figure 1:**
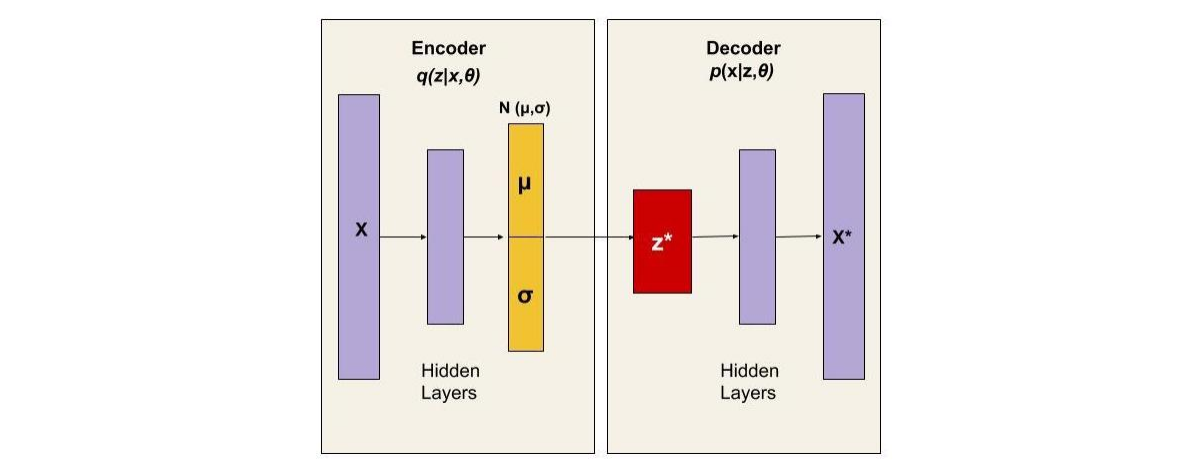
Variational auto-encoder.

The annual record of each household was defined by a set of independent equal-sized time sequences *X* = {*X*1,…,*Xt*,…,*XN*}, where each sequence is composed of 48 time units. Anomaly detection was used to decide whether the status of *Xt* was abnormal, given that all other days were known. Knowing that individuals are creatures of habit and usually have regular activity patterns, these sequences are static and periodic on a daily or weekly basis. Anomalies are the rare incidents that appear among daily patterns; therefore, identifying them through a supervised approach is challenging, as there are no labeled data available for regular and irregular activity. To answer this challenge, an unsupervised anomaly detection model should be implemented [31]. Utilizing the variational autoencoder (VAE) as a generative model can identify abnormalities by mapping the time-series data into a latent variable and reconstructing them through the latent variable [32]. In a VAE model, the encoder and decoder are defined by the probabilistic function of *q*(*z*|*x*, *Ɵ*) and *p*(*z*, *Ɵ*), respectively. The posterior distribution *q* is adapted through training and is able to map the input to a latent variable *z*. The variable *z* is assigned to a Gaussian distribution with defined parameters of mean and variance *N*(*µ_x_,σ^2^_x_*). After the encoding process, the underlying characteristic of the input is generated by sampling from the Gaussian distribution (z*) that is reconstructed during the decoding process as described in Figure 1 [32].

Due to the temporal relationship between time-series windows, the long short-term memory (LSTM)-VAE architecture was utilized to capture the latent correlation between time windows in each sequence [31]. In that way, not only are the temporal dependencies of data retained but also the information from previous time steps can be transferred to the next cell in a controlled manner. This can be done through a memory cell and 3 gates [33]. For each sequence of a time step, the hidden state (*h_t_*) is updated by the memory cell (*c_t_*). The memory cell is storing the information about the sequence that is controlled by the gate. The gates update the (*c_t_*), as the proportion of current cell input (*i_t_*), the proportion of forgetting the previous memory cell (*f_t_*), and the proportion of current cell output (*o_t_*) [33].


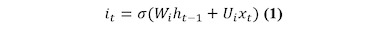



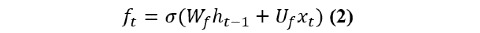



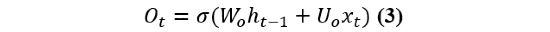



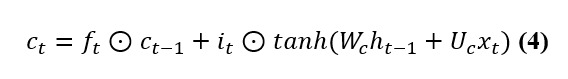



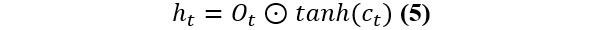


For a sample dataset X that is composed of a set of 365 independent sequences (days), each sequence was composed of 48 time intervals. The overall motion status was recorded as a unique variable in each interval. Therefore, the X dataset had the dimension of (365,48,1). Given the input X to the encoder model, the posterior distribution *q*(*z*|*x*) was approximated by feeding the LSTM’s output into 2 linear models to identify the mean and covariance of the latent variables. Then, the input of the LSTM’s hidden layers from the decoder was defined by randomly sampling from the posterior distribution *q*(*z*|*x*). The final output was defined by reconstructing the input through the random samples from the posterior distribution [34].

Based on the generative characteristics of VAE, latent variables have a key role in identifying the reconstructed data. Therefore, our objective was to model the data in such a way that the reconstructed input was similar to the original input.

This relation is represented as:


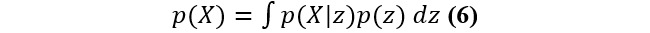


where (*X*|*z*) is the distribution of generating data from the latent variable and *p*(*z*) is the probability distribution of the latent variable.

On the other hand, the idea of VAE is to identify *p*(*z*) using *q*(*z*|*x*). However, identifying the distribution of *q*(*z*|*x*) is challenging. To this end, the variational inference model is used to approximate the *q*(*z*|*x*) distribution with simpler replacements, like standard normal or Gaussian distribution. Subsequently, the difference between the true distribution and its approximation is measured using Kullback–Leibler divergence [35].


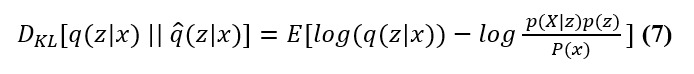


After solving this problem, the approximation error for replacing the posterior distribution with a simpler model would be defined by:


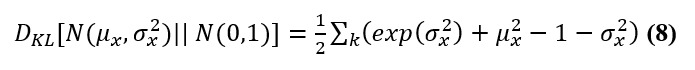


Therefore, the overall loss function is defined as 2 parts: reconstruction error between the input and output and approximation error for replacing the posterior distribution with a simpler model.


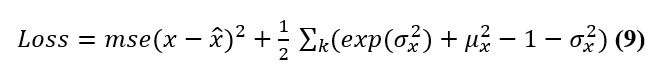


To identify the anomaly of instances, the label and score approaches are usually used. In supervised anomaly detection, the label approach is used to identify the anomalous and normal samples using the labels 1 and 0, respectively. In contrast, for unsupervised anomaly detection, the score approach is used to identify the confidence value between 0 and 1, which reflects the likelihood of an instance being anomalous [32].

Using the VAE loss function would define the anomalous sequence on the basis of the high-loss score. In that way, the distribution of scores for all the test data points would identify the median and percentile. If the score is greater or equal than “median + IQR,” it is defined as an anomalous sequence, where IQR = 75th percentile – 25th percentile.

In the training of VAE, the Keras [36] Python deep learning library was implemented. Optimization was performed using the Adam optimizer with a predefined learning rate and decay rate. The training was initialized by a mini-batch size of 7 during 200 epochs. The number of nodes for all 4 LSTM hidden layers (2 each in the encoder and decoder) was set to 100, with the *tanh* activation function for hidden layers and latent variables. We set the latent space dimensionality equal to 7 and applied an *L1* regularizer in the hidden layer of the encoder LSTM, with a weight of 0.001.

After preprocessing the data for each household, the unsupervised VAE model was implemented to identify the different regular activity patterns. From the 2018 records, the training and testing sets were randomly selected at a ratio of 80:20. The model was further validated using the records for the year 2019.

## Results

### Annual Pattern

**Figure 2:**
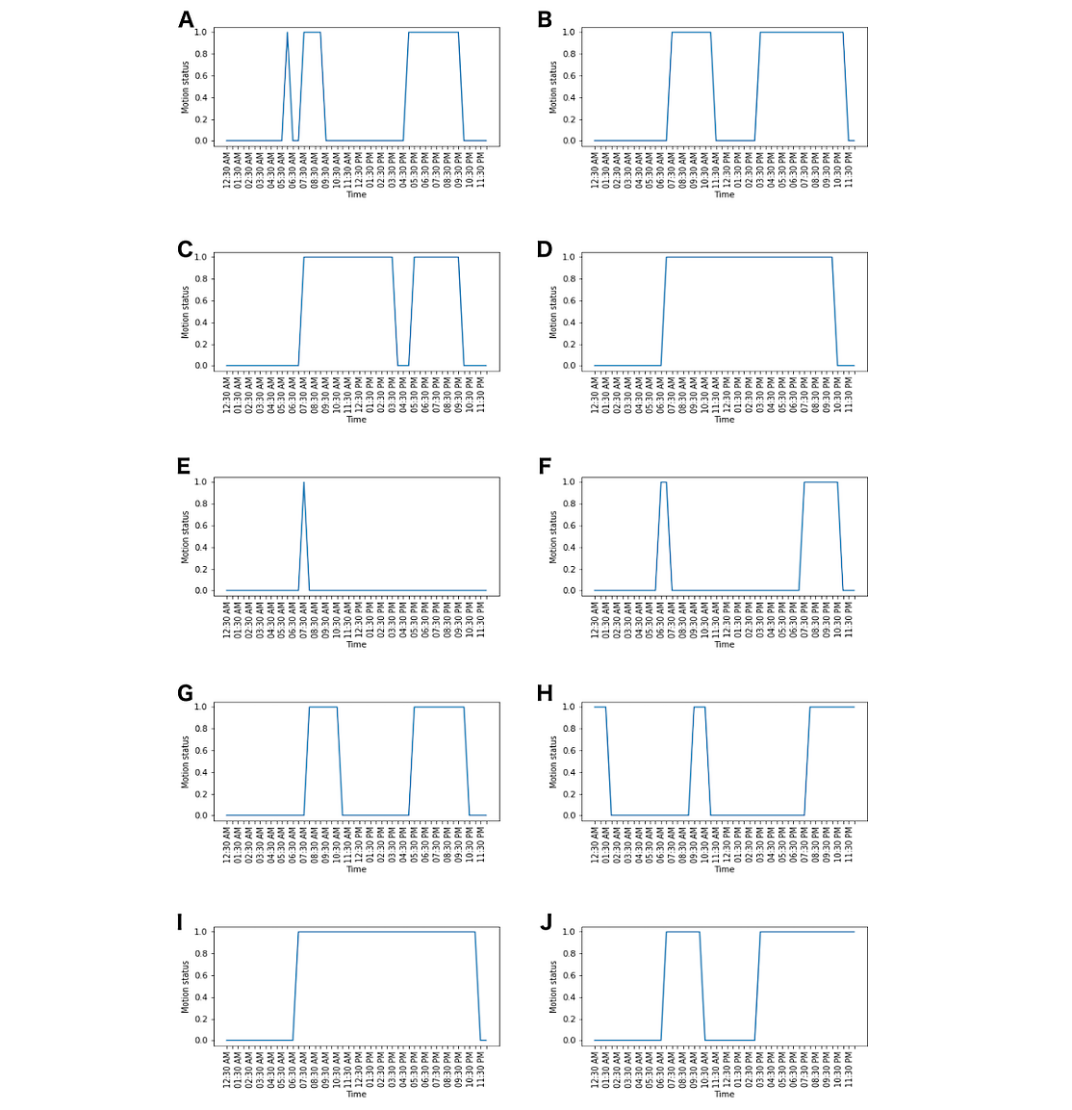
Regular activity patterns for a sample of households using variational autoencoder, which can demonstrate the diverse schedules.

The regular activity patterns for different households were identified. Figure 2 illustrates the results for the sample of 10 households. The distinct regular patterns of each household can demonstrate the diversity of the schedules.

For household A, the wake-up time was 5:30 am. The occupants left the house at 9:30 am and returned at 4:30 pm. From 4:30 pm to 10:00 pm, indoor activity was observed, while after 10:30 pm, the absence of motion could indicate sleeping time. Similar patterns were observed for households F and G. However, for household D, the wakeup time was 6:30 am, and continuous motion was observed until 10:30 pm. Households I and C also had similar patterns. The regular activity pattern for household E demonstrated that the residents spent most of their time outside of the house.

### Anomaly Detection

Anomalous activities are the rare daily patterns that differ from the regular schedule that can be defined by significant variation (median + IQR) from the regular pattern. For each household, the validation result identified the area under the curve, number of abnormal days, and average reconstruction error (loss), which are shown in Table 1.

**Table 1:** Validation result for each household based on the trained model.

Household ID	Abnormal days^a^	Total observed days	Loss^b^	AUC^c^	Abnormal weekend^d^
HH0	66	360	0.18	0.88	43
HH1	60	360	0.22	0.76	15
HH2	35	325	0.22	0.63	7
HH3	53	365	0.13	0.78	12
HH4	63	311	0.13	0.8	37
HH5	73	352	0.17	0.52	61
HH6	62	365	0.23	0.8	16
HH7	68	362	0.21	0.72	31
HH8	73	365	0.17	0.76	32
HH9	50	365	0.2	0.83	24
HH10	61	351	0.21	0.81	26
HH11	61	364	0.18	0.8	23
HH12	99	355	0.19	0.75	68
HH13	65	363	0.21	0.77	38
HH14	70	356	0.19	0.68	21
HH15	61	363	0.19	0.66	23
HH16	74	365	0.2	0.72	39
HH17	79	359	0.22	0.65	20
HH18	60	356	0.18	0.68	15
HH19	49	355	0.18	0.74	19
HH20	71	363	0.21	0.74	45
HH21	61	363	0.2	0.77	13
HH22	86	355	0.16	0.82	50
HH23	79	361	0.21	0.72	37
HH24	46	364	0.28	0.74	18
HH25	65	356	0.15	0.74	17
HH26	92	350	0.17	0.71	55
HH27	70	360	0.18	0.68	29
HH28	61	363	0.21	0.72	29

^a^Anomalous activity of users in 2019: activity that deviated from regular activity patterns defined from the 2018 data.

^b^Average error associated with reconstructing the validation records using the regular activity pattern.

^c^AUC: area under the curve. Overall compatibility of the regular activity pattern with validation records, in terms of recognizing the activation and deactivation of motion sensors at the right time slots.

^d^Total number of abnormal days that are weekend days.

**Figure 3:**
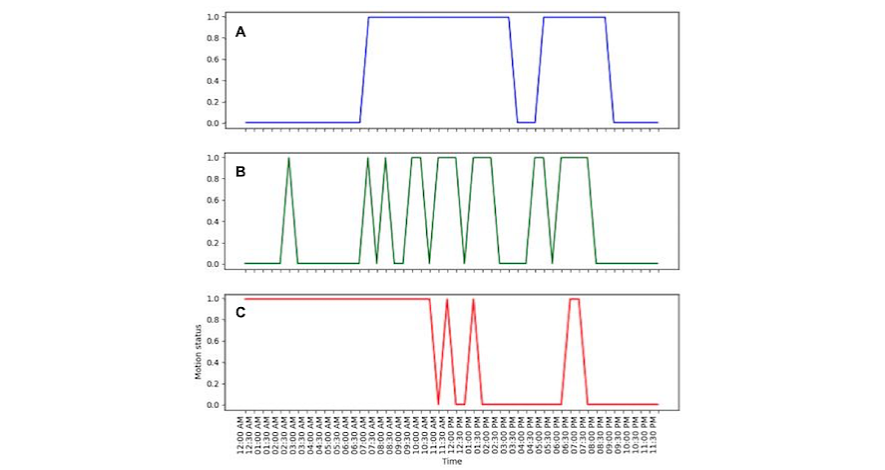
Demonstrates the regular activity pattern for a sample household (A), reconstructed normal activity (B) and anomalous activity (C).

The variation in anomalous activity and normal activity with respect to the regular schedule for a sample household is illustrated in Figure 3. In the regular activity pattern of the sample household, the wake-up time was 7:00 am, and the residents spent most of the day at home. From 4:00 pm to 5:00 pm, a lack of activity is observed in the regular pattern, which could be interpreted as either the residents are usually not inside the house or they are resting or performing other sedentary activities (watching TV). A lack of activity is also observed from 9:00 pm, which can indicate sleeping time. A similar activity pattern, with a small deviation, is observed for a normal day. For a sample anomalous day, a significant deviation from the regular pattern is observed, indicating a lack of sleep during the night and a lack of activity from 2:00 pm to 6:00 pm.

**Figure 4:**
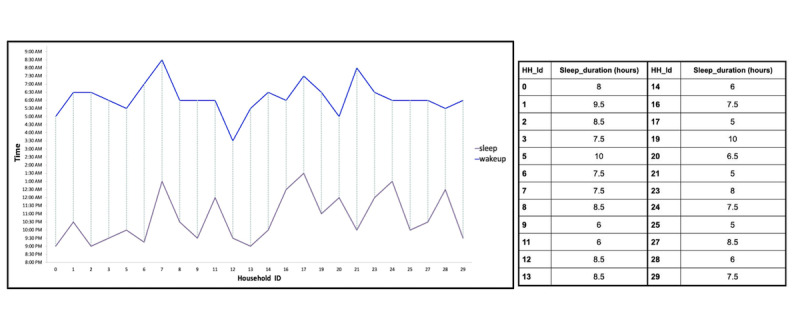
Sleep duration (dashed lines) for different households that had complete wake-up and sleeping time records. HH_Id: household ID.

In addition to anomalous behavior, other indicators such as wake-up time and sleeping time may be assessed from regular activity patterns. Figure 4 demonstrates the sleep duration of households: Their wake-up time and sleep time were recognized from the regular activity pattern (Multimedia Appendix 1).

### Weekday Patterns

**Figure 5:**
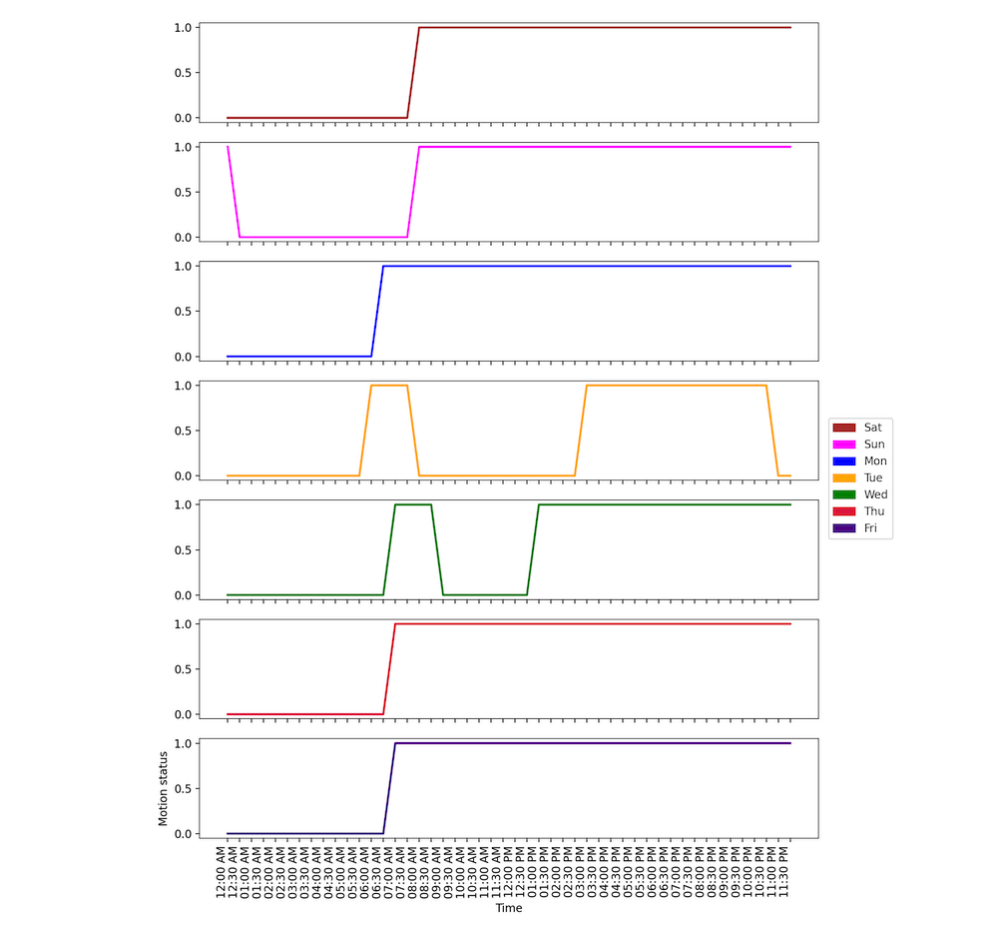
Weekday-specific activity pattern of a sample household.

To compare the variations in regular patterns based on weekdays, the annual data were divided into subsets of weekdays, and different models were defined separately. For a sample household, the most frequent weekday pattern is defined and shown in Figure 5. Individuals had similar activity patterns from Thursday to Monday and seemed to stay home all day. However, on Tuesdays and Wednesdays, the activity pattern suggests outdoor activity during the day. The wake-up time was between 6:00 am and 7:00 am on weekdays, except Tuesdays, when the wake-up time was 5:30 am. The sleeping time was between 11:00 pm and 11:30 pm except for Sunday.

**Figure 6:**
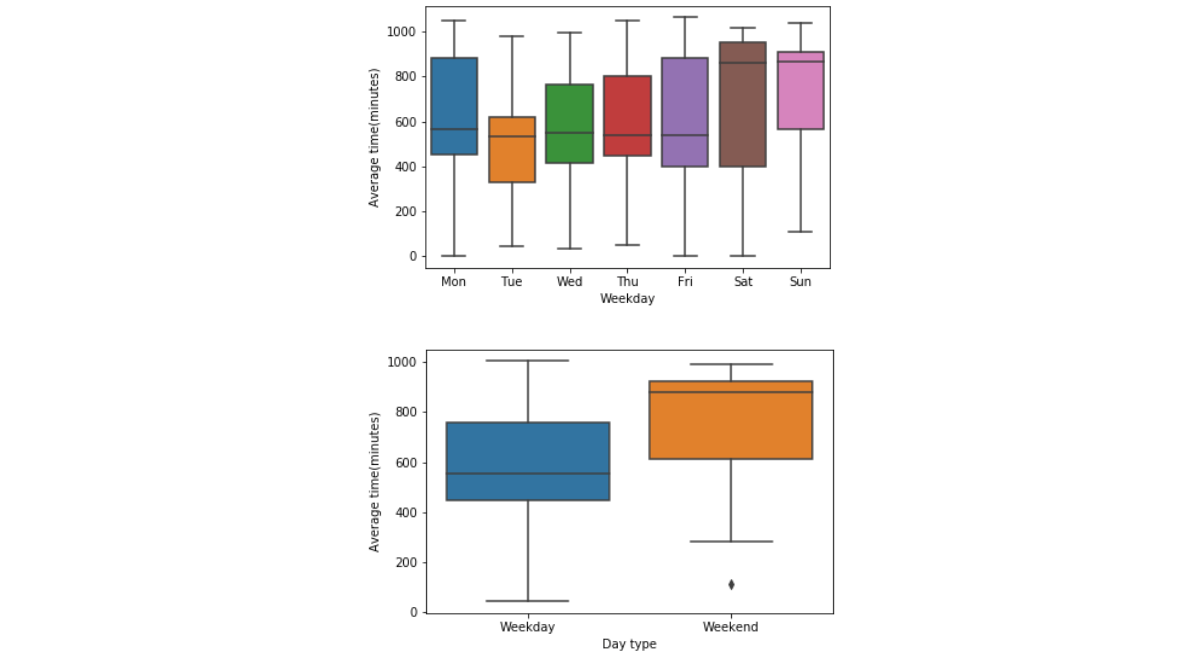
Average minutes spent at home for (A) different days of the week and by (B) day type based on the regular activity patterns of different households.

The average number of minutes that each household spent at home was compared and is shown as box plots in Figure 6, Multimedia Appendix 2, and Multimedia Appendix 3. There was a significant increase in the time spent at home throughout the weekend versus on weekdays (*P*<.05).

## Discussion

### Principal Findings

In this study, data from the ecobee smart home thermostat was used to identify the anomalous activity patterns of individuals. The large scope of these data provides mobility recognition for every 5-minute interval. The total number of sensors in each household was different, and the locations of sensors were not identified. To enhance the accuracy of motion status, we extended the time interval from 5 minutes to 30 minutes. Since there was no specified normal activity pattern available for each household, an unsupervised LSTM-VAE method was used to generate the regular activity pattern on the basis of probabilistic distribution. From Table 1, the different AUC outcomes represent the goodness of fit of the daily record to the regular activity pattern. A low AUC value represents a lack of recognition of daily activity through the model. This could be the result of a change in the schedule (HH5). In contrast, for HH0, HH4, and HH22, the high AUC value and low loss value represent a higher chance of distinguishing the daily activity pattern through the model. The number of abnormal days was defined based on the reconstruction loss threshold (Median + IQR), which would explain the households with higher average loss and lower number of abnormal days and vice versa (eg, HH2 versus HH0).

As the demographic information of users was not specified in this study, the diverse regular activity patterns observed for each household could represent the lifestyle of working professionals. However, in the case of the older population, we are expecting a more stable schedule, and any change in behavior could be considered as a sign of unexpected incidents.

In addition to recognizing abnormal daily behavior using a regular activity pattern, other insights can be determined from the model, such as assessing sleep duration and average time spent at home. As sleep duration is one the cofounding factors of individual wellbeing, it is a major concern of public health officials and health care systems to control this risk [37]. Moreover, excessive time spent inside the house can represent the severity of detachment from the natural world and sunlight, which could have detrimental impacts such as respiratory problems or seasonal affective disorder [38]. This approach has the potential, after further validation through larger studies, to provide a nonobtrusive surveillance tool to assist public health officials and governments in policy development by reducing the public health care cost and improving the quality of services in the event of an emergency by measuring indoor behavior [13-17,39,40].

However, to enhance the interpretation of the obtained results, as well as to ascertain other outcomes such as types of incidents and identify behavioral patterns in a time-based sequence of activities, more information is required. This would include demographic information about occupants, total number of residents in each household, and location of sensors (ie, labeled by type of room in the house). In addition to monitoring the activity pattern of older adults [22,24-26], this approach could also provide insights into the impact of the COVID-19 lockdown and isolation measures on daily activity patterns of households, such as sleeping time, sleeping quality, and other indoor activities.

The outputs of the models described, when trained for each household in the dataset, has potential for supporting public health surveillance [41,42]. Using the 110,000 households available in the Donate Your Data dataset [43,44], these models could generate population-level insights on sleep patterns and indoor physical activity. Data access is one of the greatest challenges in public health research [43,44], and leveraging available datasets such as the one used in this manuscript [14,15,18] allows an improved understanding of behavioral patterns without the added human resources necessary to collect subjective data from 110,000 households [45].

The same algorithms could be used to support independent living, by providing seniors and their families with an analytics layer that can be implemented on top of their ecobee smart thermostat technology, enabling family members to better understand the health of their loved ones; this has been previously undertaken by identifying activity recognition through postural transition using smartphones [5] as well as sensory-based devices [46] and fall detection using ambient sensors [16-18,40,47]. Ultimately, the utilization of IoT datasets such as the one provided by ecobee can provide measures of indoor activity, sleep duration, and sleep quality, as well as feedback to users in near real-time. It can also alert emergency response teams to adverse events such as elevated indoor temperatures during heatwave events [15]. The use of objective data as presented in this paper drives public health research away from subjective biases that challenge the domain [15].

### Conclusion

Utilizing this approach would tackle the major challenges of public health surveillance in a more applicable and efficient way. To the best of our knowledge, this is the first study that has implemented this dataset for individual health monitoring in an unsupervised manner. The results presented in this article further the development of the UbiLab Public Health Surveillance Platform, expanding on the development of algorithms for anomaly detection.

## Appendix

Multimedia Appendix 1 Average time spent home, sleeping, and awake for 30 households.

Multimedia Appendix 2 Average minutes spent at home for different days of the week, based on the regular activity model (minutes).

Multimedia Appendix 3 Comparing the average time spent home during the week versus weekend for 30 households.

## Abbreviations

AUC: area under the curve
IoT: internet of things
LSTM: long short-term memory
VAE: variational autoencoder
