# Supplementary material for: Understanding User Behavior Through the Use of Unsupervised Anomaly Detection: Proof of Concept Using Internet of Things Smart Home Thermostat Data for Improving Public Health Surveillance
Source: JMIR Mhealth Uhealth. 2020 Nov 13;8(11):e21209. doi: 10.2196/21209 (PMC7695536; doi:10.2196/21209)
Supplement: Multimedia Appendix 1 [file mhealth_v8i11e21209_app1.pdf]

Table S1. The average time spent home, sleep time, and wake-up time for 30 households is demonstrated below. The number of regular activity patterns are different for each household. For some models, the motion activity has not been observed after leaving the house. Therefore, the time spent sleeping could not be recognized.

	Minutes spending home (μ±σ)	Wake up time(μ)	Sleeping time(μ)
HH0	465±15	05 : 00	21 : 00
HH1	700±14	06 : 30	22 : 30
HH2	727±25	06 : 30	21 : 00
HH3	930	06 : 00	21 : 30
HH4	30	06 : 30	--
HH5	267±9	05 : 30	22 : 00
HH6	476±9	07 : 00	21 : 15
HH7	436±15	08 : 30	01 : 00
HH8	990	06 : 00	22 : 30
HH9	710±25	06 : 00	21 : 30
HH10	995±11	06 : 30	--
HH11	1080	06 : 00	00 : 00
HH12	442±13	03 : 30	21 : 30
HH13	333±10	05 : 30	21 : 00
HH14	591±19	06 : 30	22 : 00
HH15	707±15	07 : 00	--
HH16	480	06 : 00	00 : 30
HH17	797±22	07 : 30	01 : 30
HH18	450	06 : 00	--

HH19	987±10	06 : 30	23 : 00
HH20	566±10	05 : 00	00 : 00
HH21	628±19	08 : 00	22 : 00
HH22	90±2	--	--
HH23	650±14	06 : 30	00 : 00
HH24	325±19	06 : 00	01 : 00
HH25	945±15	06 : 00	22 : 00
HH26	145±22	06 : 00	--
HH27	811±6	06 : 00	22 : 30
HH28	730±18	05 : 30	00 : 30
HH29	900±4	06 : 00	21 : 30
