# Supplementary material for: Understanding User Behavior Through the Use of Unsupervised Anomaly Detection: Proof of Concept Using Internet of Things Smart Home Thermostat Data for Improving Public Health Surveillance
Source: JMIR Mhealth Uhealth. 2020 Nov 13;8(11):e21209. doi: 10.2196/21209 (PMC7695536; doi:10.2196/21209)
Supplement: Multimedia Appendix 2 [file mhealth_v8i11e21209_app2.pdf]

Table S2. Average minutes spent at home for different days of the week, based on the regular activity model (minutes)

	Mon	Tue	Wed	Thu	Fri	Sat	Sun
**HH0**	450 ± 8	432 ± 16	465 ± 15	448 ± 22	310 ± 29	845 ± 29	775 ± 22
**HH1**	933 ± 19	403 ± 222	875 ± 96	851 ± 67	826 ± 47	231 ± 71	430 ± 38
**HH2**	564 ± 37	260 ± 40	514 ± 33	446 ± 27	933 ± 12	932 ± 13	735 ± 42
**HH3**	903 ± 9	947 ± 18	857 ± 17	880 ± 17	920 ± 19	920 ± 14	902 ± 12
**HH4**	0	42 ± 15	33 ± 9	51 ± 18	92 ± 62	422 ± 40	466 ± 72
**HH5**	294 ± 20	305 ± 21	262 ± 13	264 ± 12	167 ± 24	188 ± 34	517 ± 195
**HH6**	470 ± 14	574 ± 26	405 ± 31	588 ± 24	441 ± 20	595 ± 55	660 ± 59
**HH7**	483 ± 41	480 ± 25	410 ± 16	480 ± 20	465 ± 27	728 ± 50	1079 ± 8
**HH8**	992 ± 7	981 ± 24	843 ± 41	1018 ± 7	707 ± 16	925 ± 39	862 ± 16
**HH9**	911 ± 21	540 ± 80	722 ± 26	523.8 ± 70	598.8 ± 93	188 ± 19.5	1037 ± 15
**HH10**	1020 ± 11	625 ± 24	778 ± 35	943 ± 72	1019 ± 6	954 ± 22	938 ± 19
**HH11**	1050	875 ± 25	996 ± 57	1050	1046 ± 10	989 ± 4	984 ± 12
**HH12**	477 ± 9	407 ± 15	428 ± 14	448 ± 8	448 ± 11	878 ± 56	909 ± 13
**HH13**	339 ± 13	299 ± 6	613 ± 70	303 ± 10	384 ± 12	775 ± 16	891 ± 26
**HH14**	423 ± 43	438 ± 21	633 ± 34	535 ± 15	522 ± 18	905 ± 20	909 ± 15
**HH15**	833 ± 18	775 ± 53	943 ± 20	806 ± 23	657 ± 29	29 ± 29	536 ± 68
**HH16**	531 ± 24	548 ± 13	495 ± 15	517 ± 20	478 ± 7	665 ± 27	850 ± 17
**HH17**	632 ± 38	283 ± 97	419 ± 25	557 ± 26	1063 ± 17	964 ± 30	1011 ± 23
**HH18**	805 ± 21	804 ± 12	266 ± 42	358 ± 48	547 ± 64	235 ± 15	348 ± 37
**HH19**	593 ± 15	561 ± 15	528 ± 21	782 ± 11	1021 ± 6	987 ± 8	959 ± 4

**HH20**	563 ± 19	545 ± 11	570 ± 17	580 ± 15	483 ± 16	982 ± 24	877 ± 13
**HH21**	507 ± 42	435 ± 18	370 ± 14	493 ± 40	808 ± 30	965 ± 12	835 ± 22
**HH22**	179 ± 4	194 ± 20	191 ± 19	122 ± 17	0	0	223 ± 26
**HH23**	653 ± 14	593 ± 14	634 ± 24	598 ± 10	621 ± 15	1015 ± 11	852 ± 23
**HH24**	127 ± 13	239 ± 20	429 ± 39	210 ± 26	336 ± 21	385 ± 28	879 ± 17
**HH25**	905 ± 15	906 ± 12	885 ± 15	923 ± 15	903 ± 10	890 ± 14	866 ± 21
**HH26**	229 ± 24	256 ± 34	101 ± 20	62 ± 7	158 ± 46	394 ± 30	418 ± 59
**HH27**	561 ± 20	605 ± 34	619 ± 21	543 ± 17	1045 ± 11	945 ± 16	946 ± 15
**HH28**	661 ± 17	524 ± 18	580 ± 17	590 ± 21	534 ± 16	988 ± 20	999 ± 21
**HH29**	900	927 ± 16	911 ± 19	908 ± 16	928 ± 16	834 ± 11	890 ± 22
