# Supplementary material for: Understanding User Behavior Through the Use of Unsupervised Anomaly Detection: Proof of Concept Using Internet of Things Smart Home Thermostat Data for Improving Public Health Surveillance
Source: JMIR Mhealth Uhealth. 2020 Nov 13;8(11):e21209. doi: 10.2196/21209 (PMC7695536; doi:10.2196/21209)
Supplement: Multimedia Appendix 3 [file mhealth_v8i11e21209_app3.pdf]

Table S3. Comparing the average spent home during the week versus weekend for 30 households.

	**Weekday (μ)** minutes	**Weekend (μ)** minutes
**HH0**	421	810
**HH1**	778	330
**HH2**	543	833
**HH3**	901	911
**HH4**	44	444
**HH5**	258	352
**HH6**	496	627
**HH7**	464	903
**HH8**	908	893
**HH9**	659	612
**HH10**	876	945
**HH11**	1003	986
**HH12**	442	893
**HH13**	387	832
**HH14**	510	906
**HH15**	803	282
**HH16**	513	757
**HH17**	590	987
**HH18**	556	291
**HH19**	696	973
**HH20**	548	929
**HH21**	522	899
